# Hospitalizations related to meningococcal infection in Spain from 1997 to 2018

**DOI:** 10.1186/s12879-021-06916-9

**Published:** 2021-12-06

**Authors:** Stefan Walter, Ruth Gil-Prieto, Mario Gil-Conesa, Gil Rodriguez-Caravaca, Jesús San Román, Angel Gil de Miguel

**Affiliations:** 1 Department of Medicine & Public Health, Universidad Rey Juan Carlos, Avda. Atenas S/N, 28922 Madrid, Spain; 2 Preventive Medicine Service, Hospital Universitario Fundación Alcorcón, Madrid, Spain; 3 PhD Student Programa de Doctorado en Ciencias de la Salud, Universidad Rey Juan Carlos, Madrid, Spain

**Keywords:** Meningococcal infection, Meningitis, Meningococcemia, Epidemiology, Vaccine

## Abstract

**Background:**

Baseline hospitalization, mortality, and in-hospital fatality rates for meningococcal infection are required to evaluate preventive interventions, such as the inclusion of the conjugated quadrivalent meningococcal vaccine and serogroup B based protein vaccines.

**Methods:**

All meningococcal infection–related hospitalizations in any diagnostic position in Spain from 1st January 1997 through 31st December 2018 were analysed. The annual hospitalization rate, mortality rate and case-fatality rate were calculated.

**Results:**

The average hospitalization rate for meningococcal infection was 1.64 (95% CI 1.61 to 1.66) hospitalizations per 100,000 inhabitants during the study period and significantly decreased from 1997 to 2018. Hospitalizations for meningococcal infection decreased significantly with age and were concentrated in children under 5 years of age (46%). The hospitalization rates reached 29 per 100,000 and 24 per 100,000 children under 1 and 2 years of age, respectively. The in-hospital case-fatality rate was 7.45% (95% CI 7.03 to 7.86). Thirty percent of the deaths occurred in children under 5 years of age, and more than half occurred in adults. The case fatality rate increased significantly with age (p < 0.001).

**Conclusion:**

It is necessary to maintain epidemiological surveillance of meningococcal infection to determine the main circulating serogroups involved, track their evolution, and evaluate preventive measures whose effectiveness must be assessed in all age groups.

## Background

Invasive meningococcal disease (IMD) is caused by *Neisseria meningitidis* infection, and its most frequent manifestation is meningitis and/or septicaemia. From 5 to 15% of the population is composed of asymptomatic nasopharyngeal carriers of *Neisseria meningitidis*, and fewer than 1% of colonized individuals develop invasive disease. Invasive meningococcal disease (IMD) has high morbidity and mortality in children and adults. IMD has significant mortality, with lethality rates of between 8 and 15% when the disease is treated but a rate of 50% when it is not treated [1]. Between 11 and 19% of patients who survive experience severe sequelae, such as hearing loss, limb amputation, and neurological complications. The disease’s significant morbidity and mortality make it a major public health problem with an estimated 500,000 annual global cases resulting in approximately 50,000 deaths per year [2].

The main causes of invasive disease are serogroups A, B, C, W, X, and Y. In the 1970s, polysaccharide vaccines against different meningococcal serogroups started to be developed. Polysaccharide vaccines, although effective and safe in the short term, have several deficiencies. They offer little or no immunogenicity in children under 2 years of age, do not generate immunological memory and are ineffective when the recipient is a carrier [3]. The development of conjugate vaccines in the 1990s spurred a breakthrough in meningococcal vaccination. These vaccines contain a polysaccharide molecule chemically conjugated to a T cell–stimulating antigen, such as a diphtheria toxoid or tetanus, increasing their immunogenicity, which has led to the achievement of immunogenicity in infants from 2 months of age, the establishment of immune memory, and the prevention of acquisition of carriage, which are important advantages that have led to herd immunity through reduced carrier status [4, 5]. Currently, monovalent conjugated vaccines against serogroups C and A and tetravalent meningococcal conjugate vaccines against A, C, W, and Y are available on the market. The latest vaccine for meningococcus is generated by reverse vaccination for serogroup B, which is immunogenic and safe to use in children older than 2 months, adolescents, and adults [6–9].

Serogroups B and C are traditionally the most frequent causes of disease in Western countries, with an important increase in serogroups W and Y occurring in the last decade [10]. However, serogroup C disease has decreased dramatically in Spain due to the incorporation of serogroup C vaccines into routine childhood vaccination schedules [11]. Meningococcal serogroups C and W account for substantial proportions of meningococcal disease in most of Africa and Latin America. Serogroup B is the predominant cause of meningococcal disease in many areas of Europe, the Americas, and the Western Pacific. Serogroup Y also causes many cases of meningococcal disease in these regions, particularly in Nordic countries. Serogroup C-conjugated vaccines were included in the routine vaccination schedule in 2001. The widespread use of serogroup B vaccines in the private vaccine market in the last decade has also affected disease in children in Spain [12]. However, since 2019, other serogroups such as W and Y have been recommended for vaccination with a conjugate tetravalent vaccine at 12 years [13]. Meningococcal vaccination in childhood in Spain is performed as follows: vaccination is applied at 4 months with conjugated MenC; depending on the vaccine used, primary vaccination with one dose (4 months) or two doses (2 and 4 months of age) may be necessary; and vaccination is applied at 12 years with MenACWY [14]. The evaluation and monitoring of meningococcal disease are necessary to determine the extent of the prevention of meningococcal disease by vaccination and to establish the burden of disease in the population.

The Spanish hospital discharge registry is a system of centralized information collection on procedures performed on patients during their hospital stay. All information is collected following the same criteria and coding. The database, called the Minimum Basic Data Set (MBDS), covers 99.5% of the Spanish population and includes more than 98% of admissions to public health system hospitals and 70% of admissions to private hospitals. It provides a complete record of all centralized hospital discharges and is not subject to the limitations of ambulatory surveillance systems [15]. The MBDS has been used for research purposes, including for epidemiological studies of different infectious diseases [16, 17].

The present retrospective epidemiological study was conducted to obtain population estimates of the hospitalization burden for meningococcal infection in the general population in Spain for a period of 22 years (1997–2018).

## Methods

For the present retrospective epidemiological study, the National Hospital Information System (MBDS) of the Spanish Ministry of Health was used as the main source of data. The collection system uses clinical codes of the Spanish version of the Clinical Modification of the International Classification of Diseases (ICD-9-CM). The system covers 98% of Spanish public hospitals and is estimated to cover 99.5% of the Spanish population [15].

All meningococcal infection–related hospitalizations of any diagnostic status for January 1st, 1997 through December 31st, 2018 were analysed. ICD-9-CM codes for meningitis infection (MI) were selected (1997–2015): 036; 036.0: meningococcal meningitis (MM); 0.36.1: meningococcal encephalitis; 036.2: meningococcaemia (MC); 0.36.3: Waterhouse-Friedrichsen syndrome due to meningococcus; 0.36.4: unspecified meningococcal carditis, meningococcal pericarditis, meningococcal endocarditis, or meningococcal myocarditis; 0.36.8: other meningococcal infections such as optic neuritis and meningococcal arthritis; and 0.36.9: unspecified meningococcal infection. For 2016–2018, the following ICD-10-CM codes were used: A39 (and subgroups) for MI and, in particular, A39.0 for MM and A39.4 for MC.

The MBDS database includes admission and discharge date, age, sex, geographic region, diagnosis, and discharge status data for all hospitalizations in the country. Hospital discharge was applied as the unit of analysis. The annual hospitalization rate and mortality rate per 100,000 individuals were calculated. For these rates, data from population municipal records were used as denominators. The in-hospital case-fatality rate (CFR), which indicates the severity of cases, was calculated by dividing the number of deaths from meningococcal infection by the total number of hospitalizations related to meningococcal infection (%). The average length of hospital stay was calculated. All analyses were stratified by age. The whole population and the population whose hospitalizations were not covered by the MBDS were assumed to have the same age, sex, and epidemiological characteristic distributions.

The national epidemiological surveillance system (RENAVE) collects cases of meningococcal disease reported in Spain and records them by serogroup. For a disease such as meningococcal disease, reporting is presumed to be of high quality. The MBDS provides relevant clinical information complementary to microbiological information and serves to measure the quality of reporting by the RENAVE network [18].

### Statistics

A chi-squared test was used to assess significant differences in proportions. Poisson regression models were used to assess differences in hospitalization rates over the study period, including by age group. For all tests, the significance level was set as p < 0.05. Statistical analyses were performed with R software (version 3.4.3).

The patient information was anonymised and deidentified prior to analysis. The local ethics committee (Comité de Ética de la Investigación de la Universidad Rey Juan Carlos) ruled that no formal ethics approval or informed consent was required for this study.

## Results

A total of 15,566 discharges were recorded with the meningococcal infection code for any diagnostic status. The mean age of the patients was 18.00 years (SD 23.95) with a median age of 6 years IQR (1 to 24). Fifty-one percent of the cases (7911) were men, and 49% (7625) were women. A total of 46% (7161) of hospitalizations involved children under 5 years of age.

**Fig. 1:**
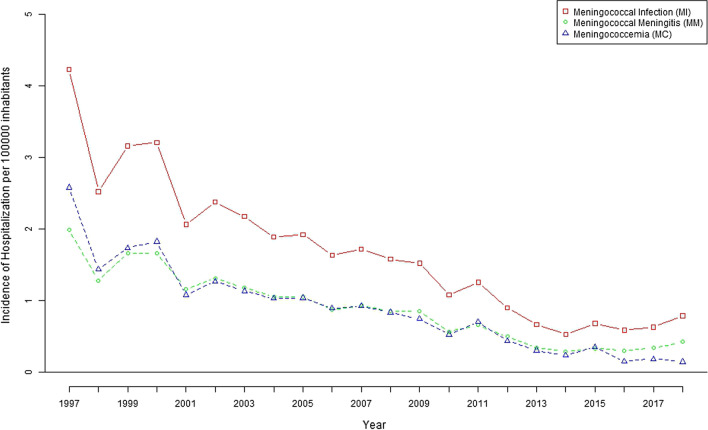
Hospitalization rate related to meningococcal infection, meningococcal meningitis and meningococcemia in Spain (1997–2018)

The hospitalization rate for meningococcal infection was 1.64 (95% CI 1.61 to 1.66) hospitalizations per 100,000 inhabitants for the study period. The rate of hospitalization for meningococcal infection decreased from 4.24 (95% CI 4.03 to 4.44) hospitalizations per 100,000 inhabitants in 1997 to 0.79 (95% CI 0.71–0.87) hospitalizations per 100,000 inhabitants in 2018 (Fig. 1). The average stay of patients with meningococcal infection was 11.71 (SD 17.70) days. Hospitalizations for meningococcal infection decreased significantly with age and were concentrated in children under 5 years of age. The hospitalization rates reached 29.01 per 100,000 children under 1 year of age and 23.89 per 100,000 children under 2 years of age (Table 1).

**Table 1:** Hospitalizations related to meningococcal infection (MI), meningococcal meningitis (MM) and meningococcemia (MC) by age group in Spain

Age (years)	Number of hospitalizations			Hospitalization rate (per 100,000) 95-CI		
	MI	MM	MC	MI	MM	MC
(0, 1)	2713	1437	1491	29.01 (27.92–30.10)	15.37 (14.58–16.16)	15.94 (15.13–16.75)
(0, 2)	4476	2237	2615	23.89 (23.19–14.59)	11.94 (11.44–12.43)	13.96 (13.42–14.49)
(0, 5)	7161	3491	4367	15.24 (14.89–15.6)	7.43 (7.19–7.68)	9.3 (9.02–9.57)
(5, 10)	1691	767	1099	3.58 (3.41–3.76)	1.63 (1.51–1.74)	2.7 (2.54–2.86)
(10, 15)	970	516	556	2.04 (1.91–2.17)	1.08 (0.99–1.18)	1.36 (1.25–1.47)
(15, 20)	1246	852	495	2.41 (2.28–2.55)	1.65 (1.54–1.76)	1.08 (0.98–1.17)
(20, 25)	642	433	246	1.05 (0.97–1.13)	0.71 (0.64–0.78)	0.44 (0.39–0.5)
(25, 30)	400	260	150	0.57 (0.51–0.62)	0.37 (0.32–0.41)	0.22 (0.19–0.26)
(30, 35)	298	172	123	0.39 (0.34–0.43)	0.22 (0.19–0.26)	0.18 (0.14–0.21)
(35, 40)	272	162	99	0.35 (0.31–0.39)	0.21 (0.17–0.24)	0.14 (0.11–0.16)
(40, 45)	262	187	70	0.35 (0.31–0.39)	0.25 (0.22–0.29)	0.11 (0.08–0.13)
(45, 50)	299	165	122	0.44 (0.39–0.49)	0.24 (0.21–0.28)	0.2 (0.17–0.24)
(50, 55)	326	196	106	0.53 (0.47–0.59)	0.32 (0.27–0.36)	0.18 (0.15–0.22)
(55, 60)	310	176	118	0.57 (0.51–0.63)	0.32 (0.28–0.37)	0.24 (0.2–0.29)
(60, 65)	326	178	107	0.67 (0.6–0.74)	0.36 (0.31–0.42)	0.25 (0.2–0.3)
(65, 70)	324	195	110	0.72 (0.64–0.79)	0.43 (0.37–0.49)	0.27 (0.21–0.32)
(70, 75)	344	184	125	0.86 (0.77–0.95)	0.46 (0.39–0.53)	0.35 (0.29–0.41)
(75, 80)	292	154	111	0.88 (0.78–0.98)	0.46 (0.39–0.54)	0.35 (0.28–0.42)
(80, 85)	255	101	98	1.05 (0.92–1.17)	0.41 (0.33–0.5)	0.43 (0.34–0.52)
(85, 120)	148	56	58	0.72 (0.61–0.84)	0.27 (0.2–0.35)	0.28 (0.2–0.36)
Total	15566	8245	8160	1.64 (1.62–1.67)	0.87 (0.85–0.89)	0.86 (0.84–0.88)

The main clinical symptoms of meningococcal infection were meningitis and meningococcaemia, and both diagnoses sometimes coexisted in the same patient. A total of 8245 patients presented meningitis, 8160 presented meningococcaemia, and 1992 presented sepsis and meningitis.

The hospitalization rate for meningococcal meningitis decreased from 1.99 (95% CI 1.85–2.13) per 100,000 inhabitants in 1997 to 0.43 (95% CI 0.37 to 0.49) in 2018, while the hospitalization rate in patients with meningococcaemia decreased from 2.58 (95% CI 2.42 to 2.74) in 1997 to 0.14 (95% CI 0.11 to 0.18) in 2018. The evolution of global hospitalization rates by year is shown in Fig. 1.

**Fig. 2:**
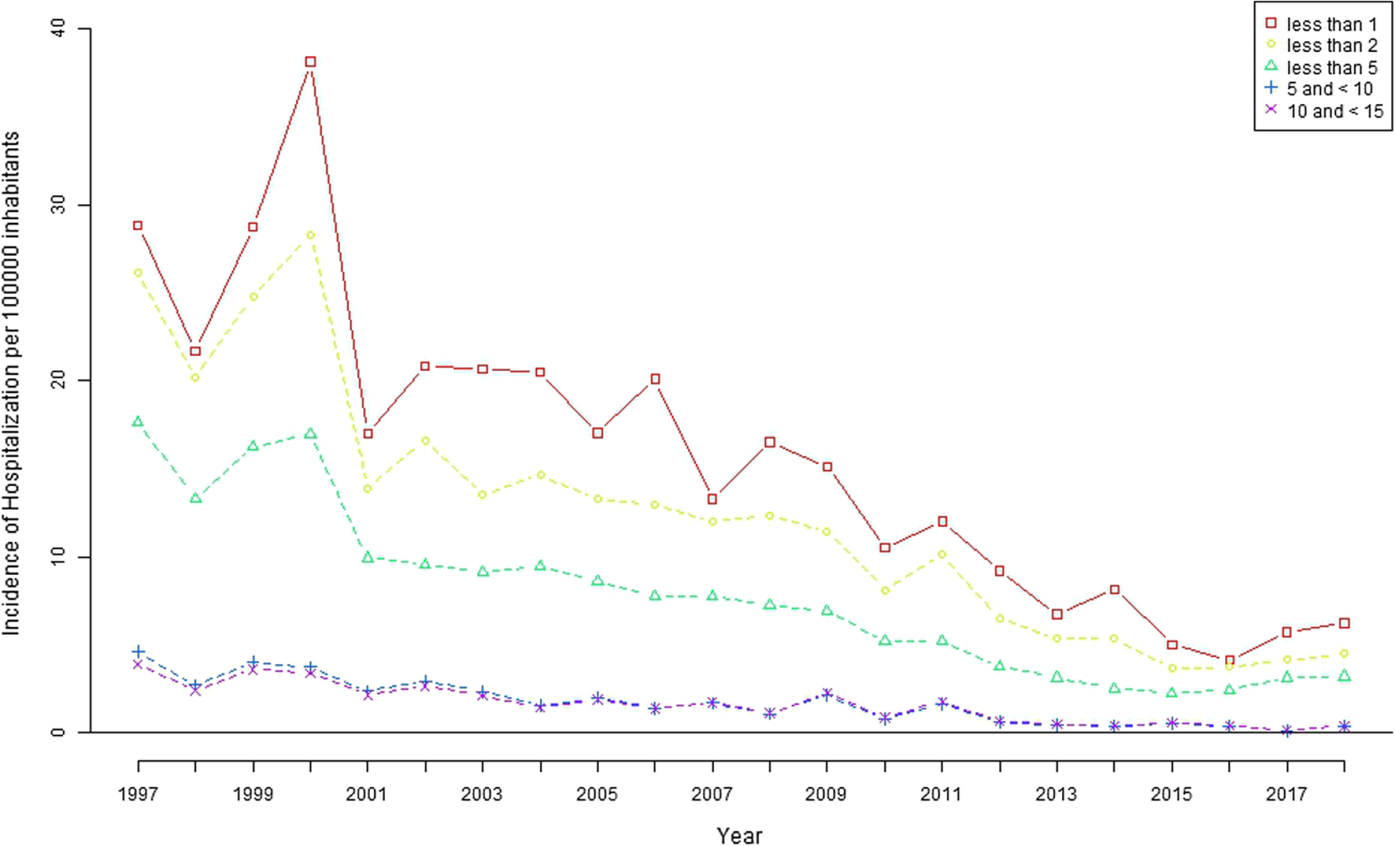
Hospitalization rate related to meningococcal meningitis by age group in Spain (1997–2018)

**Fig. 3:**
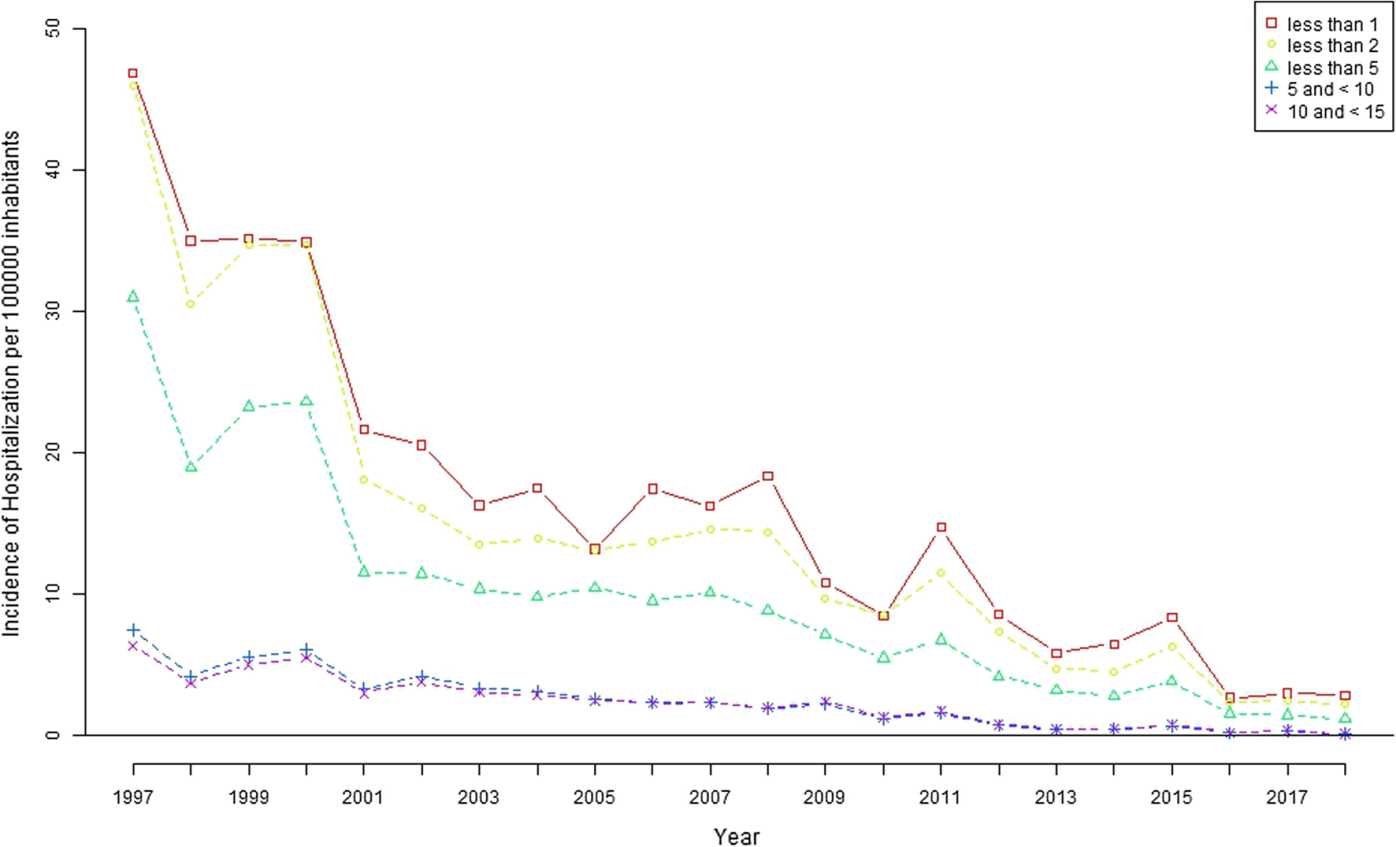
Hospitalization rate related to meningococcemia by age group in Spain (1997–2018)

These decreases in hospitalizations for meningococcal meningitis and meningococcaemia were significant for children under 5 years of age (p < 0.001 and p < 0.001, respectively) (Figs. 2 and 3).

For MM, the hospitalization rate per 100,000 inhabitants in children under 5 years of age was 17.64 (95% CI 15.72 to 19.56) in 1997 and declined to 3.19 (95% CI of 2.43 to 3.97) in 2018, with children under 1 year of age experiencing the greatest reduction. The hospitalization rate for children under 5 years of age with meningococcaemia ranged from 31.05 (95% CI 28.05 to 33.59) in 1997 to 1.16 (95% CI 0.70 to 1.63) in 2018, and children younger than 2 years of age experienced the greatest reduction.

A total of 1159 deaths occurred due to meningococcal infection in the period studied (Table 2); the overall mortality rate was 0.12 (95% CI 0.12 to 0.13) per 100,000 inhabitants, and the in-hospital case-fatality rate was 7.45% (95% CI 7.03 to 7.86). Approximately 30% of the deaths (347) occurred in children under 5 years of age, and more than half occurred in adults. Of the deceased patients, 334 had meningitis, and 785 had meningococcaemia.

**Table 2:** Mortality and case-fatality rates related to meningococcal infection (MI), meningococcal meningitis (MM) and meningococcemia (MC) by age group in Spain

Age (years)	Number of deaths			Mortality rate (per 100,000) (95% CI)			Case fatality (%) (95% CI)		
	MI	MM	MC	MI	MM	MC	MI	MM	MC
(0, 1)	136	24	106	1.45 (1.21–1.7)	0.26 (0.15–0.36)	1.13 (0.92–1.35)	5.01 (4.19–5.83)	1.67 (1.01–2.33)	7.11 (5.8–8.41)
(0, 2)	227	33	187	1.21 (1.05–1.37)	0.18 (0.12–0.24)	1 (0.85–1.14)	5.07 (4.43–5.71)	1.48 (0.98–1.97)	7.15 (6.16–8.14)
(0, 5)	347	51	293	0.74 (0.66–0.82)	0.11 (0.08–0.14)	0.62 (0.55–0.7)	4.85 (4.35–5.34)	1.46 (1.06–1.86)	6.71 (5.97–7.45)
(5, 10)	62	19	48	0.13 (0.1–0.16)	0.04 (0.02–0.06)	0.1 (0.07–0.13)	3.67 (2.77–4.56)	2.48 (1.38–3.58)	4.37 (3.16–5.58)
(10, 15)	41	12	30	0.09 (0.06–0.11)	0.03 (0.01–0.04)	0.06 (0.04–0.09)	4.23 (2.96–5.49)	2.33 (1.03–3.63)	5.4 (3.52–7.27)
(15, 20)	109	42	71	0.21 (0.17–0.25)	0.08 (0.06–0.11)	0.14 (0.11–0.17)	8.75 (7.18–10.32)	4.93 (3.48–6.38)	14.34 (11.26–17.43)
(20, 25)	59	19	41	0.1 (0.07–0.12)	0.03 (0.02–0.05)	0.07 (0.05–0.09)	9.19 (6.96–11.42)	4.39 (2.46–6.32)	16.67 (12.01–21.32)
(25, 30)	40	7	30	0.06 (0.04–0.07)	0.01 (0–0.02)	0.04 (0.03–0.06)	10 (7.06–12.94)	2.69 (0.72–4.66)	20 (13.6–26.4)
(30, 35)	31	11	19	0.04 (0.03–0.05)	0.01 (0.01–0.02)	0.02 (0.01–0.04)	10.4 (6.94–13.87)	6.4 (2.74–10.05)	15.45 (9.06–21.83)
(35, 40)	23	8	15	0.03 (0.02–0.04)	0.01 (0–0.02)	0.02 (0.01–0.03)	8.46 (5.15–11.76)	4.94 (1.6–8.27)	15.15 (8.09–22.21)
(40, 45)	27	13	11	0.04 (0.02–0.05)	0.02 (0.01–0.03)	0.01 (0.01–0.02)	10.31 (6.62–13.99)	6.95 (3.31–10.6)	15.71 (7.19–24.24)
(45, 50)	34	10	18	0.05 (0.03–0.07)	0.01 (0.01–0.02)	0.03 (0.01–0.04)	11.37 (7.77–14.97)	6.06 (2.42–9.7)	14.75 (8.46–21.05)
(50, 55)	43	13	22	0.07 (0.05–0.09)	0.02 (0.01–0.03)	0.04 (0.02–0.05)	13.19 (9.52–16.86)	6.63 (3.15–10.12)	20.75 (13.03–28.48)
(55, 60)	38	10	31	0.07 (0.05–0.09)	0.02 (0.01–0.03)	0.06 (0.04–0.08)	12.26 (8.61–15.91)	5.68 (2.26–9.1)	26.27 (18.33–34.21)
(60, 65)	44	14	30	0.09 (0.06–0.12)	0.03 (0.01–0.04)	0.06 (0.04–0.08)	13.5 (9.79–17.21)	7.87 (3.91–11.82)	28.04 (19.53–36.55)
(65, 70)	49	23	21	0.11 (0.08–0.14)	0.05 (0.03–0.07)	0.05 (0.03–0.07)	15.12 (11.22–19.02)	11.79 (7.27–16.32)	19.09 (11.75–26.44)
(70, 75)	47	16	31	0.12 (0.08–0.15)	0.04 (0.02–0.06)	0.08 (0.05–0.1)	13.66 (10.03–17.29)	8.7 (4.62–12.77)	24.8 (17.23–32.37)
(75, 80)	53	21	26	0.16 (0.12–0.2)	0.06 (0.04–0.09)	0.08 (0.05–0.11)	18.15 (13.73–22.57)	13.64 (8.22–19.06)	23.42 (15.54–31.3)
(80, 85)	68	26	31	0.28 (0.21–0.35)	0.11 (0.07–0.15)	0.13 (0.08–0.17)	26.67 (21.24–32.09)	25.74 (17.22–34.27)	31.63 (22.43–40.84)
(85, 120)	44	19	17	0.21 (0.15–0.28)	0.09 (0.05–0.13)	0.08 (0.04–0.12)	29.73 (22.37–37.09)	33.93 (21.53–46.33)	29.31 (17.6–41.03)
Total	1159	334	785	0.12 (0.12–0.13)	0.04 (0.03–0.04)	0.08 (0.08–0.09)	7.45 (7.03–7.86)	4.01 (3.62–4.48)	9.62 (8.98–10.26)

The case fatality rate increased significantly with age (p < 0.05) for all meningococcal infections and when studying meningitis and meningococcaemia separately. The rates were 4.85%, 1.46%, and 6.71% for MI, MM, and MC in children under 5 years of age, respectively, and they were close to 30% in individuals over 85 years of age. The case-fatality rate was significantly higher when the infection involved sepsis (p < 0.001).

## Discussion

This retrospective study reports the epidemiology of meningococcal infections in Spain over a period of 22 years. At this time, meningococcal disease has significantly decreased in incidence but continues to have important relevance to public health. In the period studied, 15,566 hospitalizations for meningococcal infection occurred with a total of 1159 deaths, 347 of which occurred in children under 5 years of age.

In addition to its clinical burden, meningococcal disease is associated with a significant economic burden on the public health system in the short and long term [19]. In both children and elderly people, attention to the prevention of IMD should be strengthened due to the significantly higher rate of hospitalizations and deaths occurring in these age groups [20].

The average duration of hospitalization related to IMD was found to be 12 days in our study, in line with studies from neighbouring countries (e.g., 13 days in Germany [21]). The clinical distribution is also similar with a similar number of meningitis and sepsis cases and their coexistence observed in 13% of those hospitalized. In Germany, 38% of all cases presented had only meningitis, 35% had only sepsis, 16% had both, and 11% had other IMDs. IMD leads to serious complications and sequelae and is associated with extensive costs and an increased use of health resources in Germany, especially in the first year after the diagnosis of IMD and due to hospitalization related to IMD [21].

In our study, the case-fatality rate was recorded as 7%, which is somewhat higher than that observed in the United Kingdom, where the case-fatality rate is 4.46% [22]. Older age was significantly associated with higher lethality due to IMD, ranging from 5% in children younger than 5 years of age to 30% in people older than 85 years of age. This finding is consistent with previous results obtained in Spain [5] and by studies conducted in other European countries and around the world [23, 24].

Rates of hospitalization and mortality decreased in the period studied, especially from 2000 with the introduction of the conjugated vaccine for serogroup C as observed in Spain [11] and nearby countries [23, 25]. In the present study, we observed this global decrease, which lowered the MI rate from 4.24 in 1997 to 0.79 in 2018. The population most benefiting from the introduction of the vaccine is that under 5 years of age, who have seen their MI rate drop from 44.1 in 1997 to 5.4 in 2018.

At this time, in Europe, the incidence of invasive meningococcal disease is < 1 cases per 100,000 people per year in countries with implemented epidemiological surveillance [26]. The countries that have implemented routine vaccination with serogroup C conjugate vaccine have the highest IMD burden due to serogroup B. Thus, for example, in Italy’s National Surveillance of Invasive Bacterial Diseases, serogroup B accounted for 64.9% of samples serotyped in 2011 [27], and in the UK, serogroup B meningococci were responsible for 87.33% of cases [22].

The total incidence of invasive meningococcal disease (IMD) in Europe has been decreasing in recent years; however, an increasing incidence due to serogroup W (MenW) has been reported in some European countries, mainly in England, the Netherlands, Switzerland, and Sweden. Serogroup W is more frequently found in older age groups, while the proportion in children (< 15 years) is lower than that of other age groups [28].

In addition, the unplanned but beneficial reduction in the carrier status of vaccine serogroups was demonstrated after the implementation of the vaccination campaigns of MenAfriVac and MCC, both with conjugated vaccines [29–31]. The application of MenAfriVac led to a notable decrease or elimination of the transport of serogroup A in all age groups. Similarly, three European countries have achieved herd immunity as observed by the rate of MCC in unvaccinated age groups, suggesting a tangible impact of the vaccine [29–31].

In 2018, the Global Meningococcal Initiative, whose objective was to prevent IMD worldwide through education, research, and cooperation, focused its annual meeting on the evolution of epidemiology, surveillance, and protection against IMD worldwide with an emphasis on resistance to antibiotics and the protection of high-risk populations. The Global Meningococcal Initiative is composed of a multidisciplinary group of scientists and doctors representing institutions from several continents. Given that the incidence and prevalence of meningococcal disease continuously vary both geographically and temporally and as surveillance systems differ throughout the world, the true burden of IMD remains unknown. The predominant serogroups are B and C, followed by A, but in recent years, cases attributable to serogroups W, X, and Y have emerged. Vaccination and antibiotic prophylaxis are the pillars of IMD prevention. Experiences around the world support the use of conjugate vaccines given their ability to act on carriers, and new protein vaccines show great promise. The application of protection strategies to high-risk groups, including people with asplenia, complement deficiencies, or human immunodeficiency virus; laboratory workers; and people receiving eculizumab, as well as to control antibiotic resistance, can prevent outbreaks. However, there is evidence that a reduction in antibiotic susceptibility is becoming more prevalent worldwide [26, 32].

The Spanish Association of Paediatrics recommends that meningococcal vaccine against serogroup B, with a 2 + 1 schedule, be included in the routine schedule in addition to the inclusion of the conjugated quadrivalent meningococcal vaccine (MenACWY) at 12 years of age with follow-up at 18 years. The vaccine advisory committee of the Spanish Paediatric Association recommends that the vaccine also be used at 12 months of age, replacing MenC. Likewise, the vaccine is recommended for those older than 6 weeks who have risk factors or who travel to countries with a high incidence of these serogroups [33]. These recommendations are in line with those of surrounding countries. For example, in France, where the incidence of invasive meningococcal disease is approximately 1/100,000, the schedule still includes only a C-conjugate meningococcal vaccine, and it has good adherence in infants but not in adolescents and young adults [34].

In Europe, it is time to consider not only national epidemiology but also trends in neighbouring countries. The increase in cases of Group W encourages a shift from the use of the C vaccine to the adoption of the ACWY vaccine in both infants and adolescents. It is also time to protect babies with vaccine B [14].

This study has some limitations related to the use of a national hospital database. The reliability of hospital surveillance depends on the quality of discharge reports and clinical histories and on the quality of the coding process [15]. The MBDS does not provide information on laboratory confirmation or on the serogroup causing infections. However, we assumed that a differential diagnosis of severe meningococcal infection had been confirmed, since it is a reportable disease in Spain. Additionally, we acknowledge the change in ICD codification, whose impact on the classification of MI, MM, and MC cannot be evaluated.

Despite its limitations, the MBDS is a tool that has proven useful for studies of disease burden in Spain and that has been used for this purpose previously, which makes it a reliable source of good quality that is collected in a standardized way in virtually the entire country [16]. In addition, the nature of IMD as such a serious disease with very high rates of hospitalization makes it possible to use hospitalization rates as a means to monitor the incidence of the disease.

## Conclusions

The information presented in this article will allow for the comparison of new data after the implementation of vaccination with the ACWY conjugate vaccine at 12 years of age and the comparison of the different strategies used by different regions at 12 months of age. It is necessary to maintain epidemiological surveillance to determine the main circulating serotypes, track their evolution, and establish preventive measures to be implemented whose effectiveness can be measured in all age groups.

## Data Availability

All of the data generated or analysed during this study are included in this published article and are available on request at https://pestadistico.inteligenciadegestion.mscbs.es/publicoSNS/S/rae-cmbd.
